# Study on the mechanism of cadmium chloride pollution accelerating skin tissue metabolism disorder, aging and inhibiting hair regeneration

**DOI:** 10.3389/fpubh.2022.1035301

**Published:** 2022-10-19

**Authors:** Weibin Du, Yi Dong, Zhenwei Wang, Sai Yao, Meijiao Wang, Jinjun Ji, Hongfeng Ruan, Renfu Quan

**Affiliations:** ^1^Research Institute of Orthopedics, The Affiliated Jiangnan Hospital of Zhejiang Chinese Medical University, Hangzhou, China; ^2^Hangzhou Xiaoshan Hospital of Traditional Chinese Medicine, Hangzhou, China; ^3^Shaoxing Traditional Chinese Medicine Hospital Affiliated to Zhejiang Chinese Medical University, Shaoxing, China; ^4^Institute of Orthopedics and Traumatology, The First Affiliated Hospital of Zhejiang Chinese Medical University, Hangzhou, China; ^5^The First Clinical College of Zhejiang Chinese Medical University, Hangzhou, China; ^6^School of Basic Medicine Sciences, Zhejiang Chinese Medical University, Hangzhou, China

**Keywords:** skin, cadmium pollution, metabolism, aging, hair regeneration

## Abstract

Drinking water contaminated by Cd2^+^ is one of the main pathways for Cd to enter the body. The skin barrier is destroyed when the skin is contaminated by environmental Cd2^+^, however, the detailed mechanism by which Cd2^+^ induces skin metabolic disorder, and senescence and affects hair regeneration is not completely understood. In this study, 18 C57BL/6 mice were randomly divided into a Control group, a Low-dose group, and a High-dose group with 6 mice in each group, and intragastrically administered with different concentrations of cadmium chloride once a day, respectively. After 1 month of intervention, the skin tissues on the back of mice were collected for non-targeted metabolomics analysis, and the related proteins were detected by immunofluorescence assay. Non-targeted metabolomics analysis result showed that compared with the Control group, there were 29 different metabolites, mainly including lysophospholipids, fatty acids, and bile acids, in the Low-dose group, and 39 differential metabolites in the High-dose group, in addition to the above compounds, there were more amino acid compounds, and most of the metabolites had a reduced response after administration. Immunofluorescence assay result showed that the higher the concentration of cadmium chloride led to the more obvious the proliferation inhibition and apoptosis promotion effects of skin cells, and the more significant damage to hair follicle stem cells. Thus, our findings demonstrate that cadmium chloride pollution can accelerate skin metabolism disorder, and aging and impair hair regeneration.

## Introduction

Cadmium (Cd) is a kind of toxic heavy metal, which is distributed in the natural environment due to its extensive application in the industry (1), and it can also be discharged into the air through diesel exhaust. Cd^2+^ is one of the major toxic substances detected in the environment. Cd^2+^ pollution is still increasing in the world, and it has become a major ecological problem faced by developing countries such as China (2). Even low doses of Cd^2+^ are toxic, with a long half-life of up to several decades (3). It can accumulate in the body with long-term dietary intake, resulting in and developing a range of chronic diseases, causing serious harm to human health. Chronic low doses of Cd^2+^ (2 mg/kg) have been shown to induce a variety of pathological conditions, disrupting renal metabolism in rats even when exposed to very low doses of Cd^2+^ (0.7 mg/kg) (4). Cd^2+^ (2 mg/kg) exposure has been shown to cause fertility damage and testicular cell apoptosis in male C57BL/6J mice (5). Cd^2+^ (7 mg/kg) exposure can cause liver lipid peroxidation and liver injury in male CD mice (6). In short, it affects many tissues and organs, including the liver, kidneys, brain, testis, and thymus, both *in vivo* and *in vitro* (7). Thomas et al. found that Cd concentrations in the urine of people with long-term Cd^2+^ exposure were comparable to the levels of kidney and bone effects found in other populations (3). It is well known that Cd^2+^ is a carcinogen, and Cd^2+^ exposure can promote the development of a variety of malignant tumors, including leukemia and lung cancer.

The skin is the largest organ of the human body and is a complex human protective barrier, protecting the human body from external invasion and moisture loss through the physical and chemical barriers (8). Skin inflammation is attributed to the derangement of the epidermal barrier function (9). Studies have shown that disruption of the skin barrier can lead to the development of a variety of diseases, including atopic dermatitis, contact dermatitis, pruritus, and Sjögren syndrome, as well as systemic diseases that may be associated with aging (10, 11). When the skin is contaminated by environmental Cd^2+^, the barrier is destroyed, accompanied by apoptosis, DNA damage, and lipid oxidation. At the same time, skin antioxidant enzymes such as glutathione peroxidase and methionine sulfoxide reductase are also destroyed (2). Ceramide also plays a crucial role in maintaining the skin barrier function (12). The cuticle is the skin's main mechanical barrier, formed by the multiple actions of lipids and composed of ceramide, cholesterol, and fatty acids. All three components play critical roles in skin integrity, particularly ceramide, which is essential for maintaining epidermal homeostasis, and skin ceramide levels decline with skin aging (11). The main mechanism by which Cd^2+^ exerts its deleterious effects is through the generation of oxidative stress leading to skin senescence (13), thereby disrupting skin homeostasis. It has also been reported in the literature that disruption of skin barrier function may also be associated with autophagy (14). Although these pathways may provide useful information, the mechanisms of skin barrier disruption by Cd^2+^ are still poorly understood.

Drinking water contaminated with Cd^2+^ is one of the main pathways that Cd^2+^ enters the body (15). Specifically, the Environmental Protection Agency (EPA) mandate that the concentration limit for Cd in drinking water should be as low as 5 ppb (16). However, emerging evidence suggests that the Cd^2+^ concentration in the contaminated waters is much higher than this value (17). Some studies have shown that heavy metals have potentially harmful effects on the skin, but there are few works of literature about the effects of heavy metals on the skin, and the mechanism of Cd^2+^-induced skin tissue damage remains obscure. This study aimed to investigate how Cd^2+^ induces metabolic disorder and senescence in mouse skin and to explore the effects of Cd^2+^ on cell proliferation, apoptosis, and hair regeneration.

## Methods

### Experimental animals

Eighteen male C57BL/6 mice (5 weeks old, 10–16 g body weight) were provided by the animal experiments center of Zhejiang Chinese Medical University [Grade SPF, SCXK (Shanghai)]. All animal procedures was approved by the Zhejiang Chinese Medical University Animal Ethics Committee (No. IACUC-20211227-03) and followed the National Institutes of Health and Animal Research Committee guidelines for Animal Research.

### Grouping and processing

Eighteen male C57BL/6 mice were divided into 3 groups (*n* = 6 per group): Control group, Low-dose group, and High-dose group. The low-dose group and the high-dose group were intragastrically administered with cadmium chloride (CdCl_2_) once daily (2 and 7 mg/kg/time, respectively). And the mice in the Control group were intragastrically administered with equal volumes of water (100 μL). After 4 weeks treatment, the mice were sacrificed, and the hairs on the back were removed (first the long hairs on the back were cut off, then the remaining hairs were shaved off with a razor), and the skin on the back was taken for further examination.

### LC-MS sample preparation

The skin tissue was cut into pieces, and methanol (1 mg/10 μL) was added at a ratio of 1:10 (W/V). Two magnetic beads were added, homogenized at 60 Hz for 5 min, then ultrasound was continued for 20 min, centrifugation at 13,000 r/min for 10 min, and 400 μL of the supernatant was dried with nitrogen. The supernatant was redissolved in 50 μL 70% methanol and centrifuged at 13,000 r/min for 15 min after 2 min of vortexing. The supernatant was transferred to the injection vial for testing. Some of the supernatants after centrifugation by equal absorption of homogenate, nitrogen blow-drying, re-dissolving, and preparing quality control (QC) samples. The samples were analyzed by UHPLC-QTOF/MS under liquid conditions.

### UHPLC-QTOF/MS analysis

#### Chromatography

Chromatographic separation was performed on an ExionLC system (AB Sciex, Foster City, CA, USA). A Waters Acquity HSS T3 column (2.1 × 150 mm, 1.7 μm) was applied at the temperature of 35°C. The mobile phase A was water with 0.1% formic acid (v/v), and B was acetonitrile. The gradient was optimized as follows: 0–5 min from 3 to 8% B, 5–11 min from 8 to 30% B, 11–20 min from 30 to 80% B, 20–21 min from 80 to 95% B, 21–27 min at 95% B, then back to the initial ratio of 3% B and maintained with additional 6 min for re-equilibration. The injection volume of all samples was 2 μL.

#### Mass spectrometry

To provide high-resolution detection, a 5600 Q-TOF mass spectrometer (AB Sciex, Foster City, CA, USA) equipped with an electrospray ionization source (Turbo Ionspray) was applied. MS detection was implemented both in negative and positive ion mode with the mass rang at m/z 100–1,250. The parameters of the mass spectrometer were summarized as follows: gas 1 and gas 2, 45 psi; curtain gas, 35 psi. Heat block temperature, 550°C; ion spray voltage, −4.5kV in negative mode and 5.5 kV in positive; declustering potential, 50 V; collision energy, ±35 V; and the collision energy spread (CES) was ±15 V. To monitor the reproducibility and stability of the acquisition system, QC samples were prepared by pooling small aliquots of each sample. The QC specimens were analyzed every five samples throughout the whole analysis procedure.

#### Data processing

The original map was extracted by SCIEX OS Analytics and the data matrix was transformed, including mass-to-charge ratio (m/z), retention time (RT), and intensity. All data were normalized with total peak area to generate an excel table for analysis of the subsequent metabolome. To reduce the signal interference caused by accidental error, the variables with RSD ≥ 40% in QC are eliminated in excel first.

The excel file was imported into SIMCA 14.1 (Umetrics, Umeå, Sweden) software for multivariate statistical analysis. The whole distribution of samples was observed by principal component analysis (PCA). In addition, the consistency of the samples within the group was analyzed by PCA-class analysis. Generally, under one principal component, when the samples fell outside the “2-std. dev.” line, it is considered that the sample is abnormal data, and the sample data should be eliminated before the follow-up analysis.

#### Analysis of differential metabolites and metabolic pathways

OPLS-DA permutation test was used to statistically analyze the validity of the OPLS-DA model. When *Q*^2^ intersects the Y-axis, the model was validated, and then differential metabolites were screened. Based on this model, the different variables were screened according to the variable projection importance index (VIP value), and the variable with VIP > 1 was considered a meaningful variable that caused the difference. Furthermore, the partial correlation coefficient was used to screen the variables that had a great influence on the OPLS-DA model. Finally, Mann-Whitney Test was performed on the selected variables, and *P*-value < 0.05 was the significant difference variable. S-plot and volcano plots are produced and visually reflect the contribution of each variable to the differential grouping. Potential markers were identified by HMDB (http://www.hmdb.ca/) and LIPID MAPS (https://www.lipidmaps.org/). The differential metabolite heatmap was made to directly reflect the response degree of the compounds after administration. Based on the results of screening and identification of significantly different metabolites, the compound name results of each group were introduced into Metabo Analyst 5.0 (http://www.metaboanalyst.ca/) for metabolic pathway analysis.

### Immunofluorescence assay

Skin tissue samples were subjected to a frozen section, rinsed in PBS, fixed in 4% PFA solution, removal of endogenous Peroxidase, BSA blocking, incubation with target primary antibody (1:500), 4°C overnight, and PBS rinsing 3 times followed by incubation of secondary antibodies at room temperature, the nuclei were counterstained with DAPI. After sealing the slices, the images were observed and collected under fluorescence microscope, and then processed and compared with Image-J software.

### Statistical analysis

All of the experimental results were expressed as the mean ± SD (standard deviation). All statistical analyses were performed using SPSS 21.0 software. The significance of differences between groups was determined by 2-tailed unpaired Student's *t*-test or one-way ANOVA with Dunnett's *post-hoc* test when samples were not distributed normally. A value of *p* < 0.05 was considered to be statistically significant.

## Results

### Repeatability and stability of the UHPLC-QTOF/MS method

**Figure 1:**
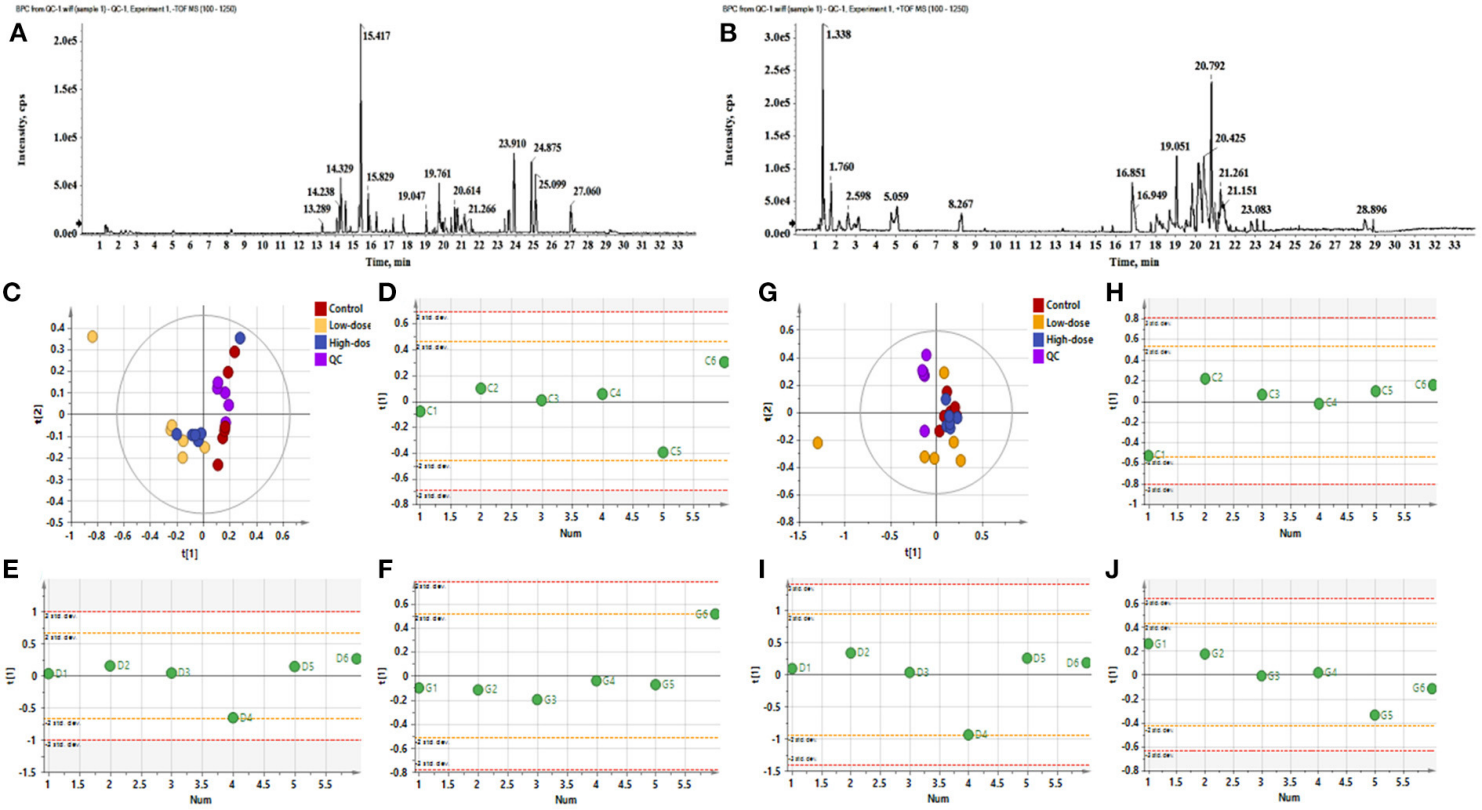
The BPC diagram of QC samples in positive and negative ion mode. **(A)** Negative ion mode, **(B)** Positive ion mode. **(C–F)** PCA analysis of negative ions. **(C)** PCA analysis of all samples (R^2^X 0.756, *Q*^2^ 0.396), **(D)** PCA-class analysis of Control group, **(E)** PCA-class analysis of Low-dose group, **(F)** PCA-class analysis of High-dose group. **(G–J)** PCA analysis of positive ions. **(G)** PCA analysis of all samples (R^2^X 0.566, *Q*^2^ 0.0992), **(H)** PCA-class analysis of Control group, **(I)** PCA-class analysis of Low-dose group, **(J)** PCA-class analysis of High-dose group.

The data in Figures 1A,B are QC Base Peak Chromatograms (BPC) in positive and negative ion modes. The base peak diagram is a continuous representation of the strongest ionic strength at each time point, which includes the ionic strength and the retention time of the ions in the chromatography. The PCA plots of all samples in positive and negative ion mode are shown in Figures 1C,G, respectively, showing that the QC samples clustered more closely, indicating good stability and reproducibility of this experiment. The consistency of the within-group samples was analyzed by PCA-Class analysis. From the results of Figures 1C–J, it can be seen that although some points negative ions D4, G6, positive ions C1, D4 fall on the 2std line, they do not exceed it, therefore, all data will be retained.

### Cd^2+^ exposure causes difference in metabolites in mouse skin tissue

#### Low-dose Cd^2+^ exposure results in 29 differential metabolites in mouse skin tissues

**Figure 2:**
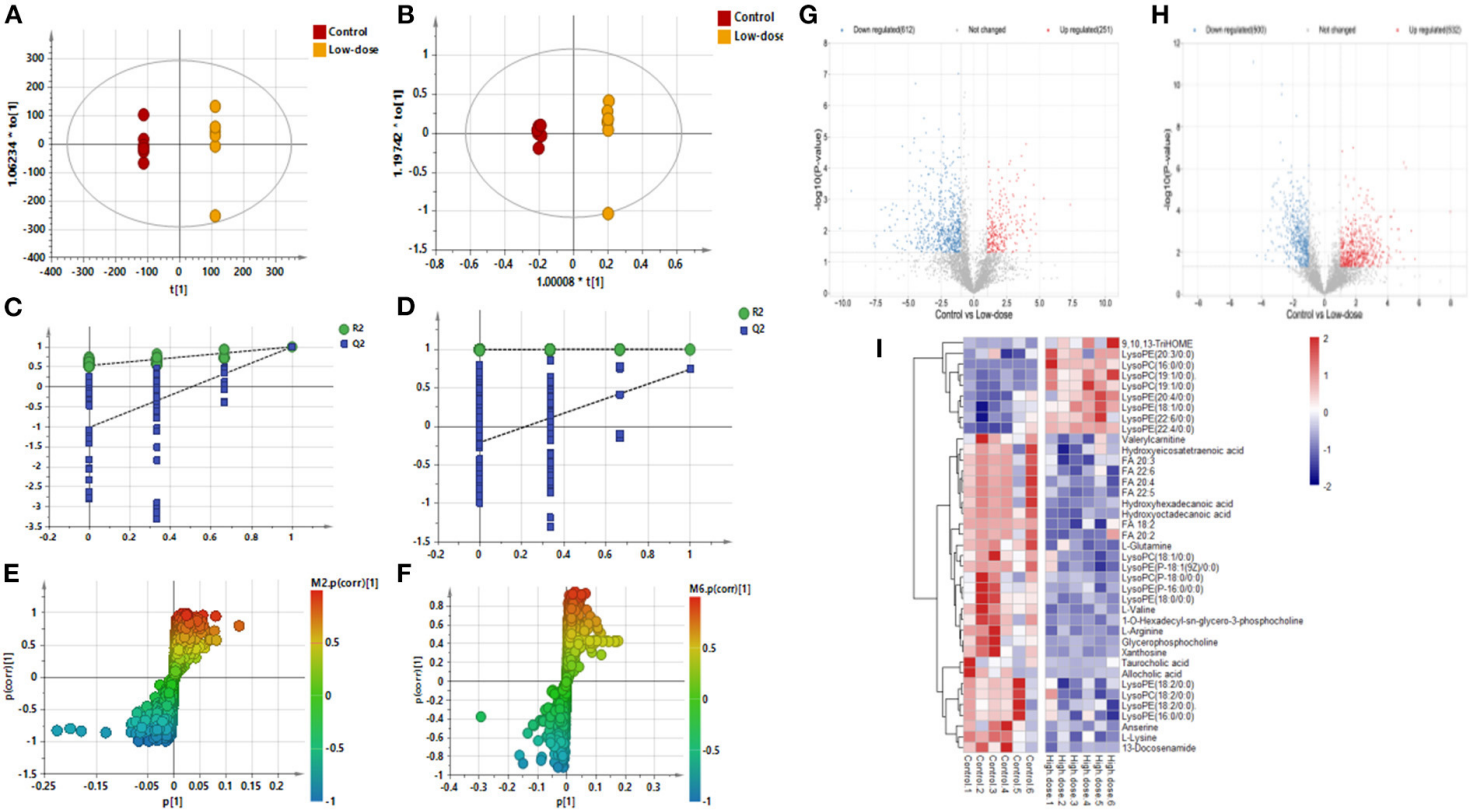
OPLS-DA: Control vs. Low-dose. **(A)** Negative ions (R^2^X 0.915, R^2^Y 1.000, Q^2^ 0.992), **(B)** Positive ions (R^2^X 0.857, R^2^Y 0.999, Q^2^ 0.738), **(C)** Negative ion replacement test, **(D)** Positive ion replacement test. **(E)** Negative ion S-plot, **(F)** Positive ion S-plot. **(G,H)** Volcano diagram. **(G)** Negative ions, **(H)** Positive ions. ① Fold change (M/C) > 2 or <0.5, and ② *P* <0.05 was the effective variable. Blue means down, red means up. **(I)** Control vs. Low-dose differential metabolite heat map. The darker the red, the higher the response, and the darker the blue, the lower the response. It can be seen that the response of most compounds decreased significantly after administration of low concentrations.

Figures 2A–F shows the OPLS-DA of Control *vs*. Low-dose, showing that the two groups are distinguished and the model is valid. Figures 2G,H is a volcano plot of Control vs. Low-dose, with red indicating variables upregulated after CdCl_2_ gavage, blue indicating variables downregulated after CdCl_2_ gavage, and gray indicating variables that did not differ. A total of 29 different metabolites of Control vs. Low-dose were screened and identified, which were primarily lysophospholipids, fatty acids, and bile acids. And most metabolites showed a reduced response after administration, the results are shown in Table 1. The heat map can be more intuitive to observe the difference between the two groups of variable intensity responses, the results are shown in Figure 2I.

**Table 1:** Basic information of differential metabolites in Control vs. Low-dose.

**Metabolites**	**Formula**	**m/z**	**Rt min**	**VIP**	***p* (corr)**	***p*-value**	**Fold**
N-Glycolylneuraminic acid	C_11_H_19_NO_10_	324.0936	1.3	3.00	−0.91	0.0022	0.27
Inosine	C_10_H_12_N_4_O_5_	267.0735	3.76	2.87	−0.84	0.0043	0.32
Valerylcarnitine	C_12_H_23_NO_4_	246.1700	9.66	1.08	−0.76	0.0022	0.44
Taurallocholic acid	C_26_H_45_NO_7_S	516.2990	14.01	1.06	−0.39	0.0411	0.13
Taurocholic acid	C_26_H_45_NO_7_S	533.3255	15.19	3.86	−0.47	0.0152	0.07
LysoPE(18:2/0:0)	C_23_H_44_NO_7_P	476.2783	19.77	2.04	−0.63	0.0152	0.66
LysoPE(20:4/0:0)	C_25_H_44_NO_7_P	502.2928	19.82	4.52	−0.79	0.0022	0.73
LysoPC(18:2/0:0)	C_26_H_50_NO_7_P	542.3217	20.14	1.19	−0.77	0.0087	0.65
LysoPE(18:1/0:0)	C_23_H_46_NO_7_P	478.2939	20.87	2.23	0.73	0.0087	1.19
LysoPC(18:1/0:0)	C_26_H_52_NO_7_P	566.3463	20.98	1.59	−0.63	0.0022	0.72
LysoPE(22:4/0:0)	C_27_H_48_NO_7_P	530.3241	21.13	2.05	0.65	0.0411	2.06
1-O-Hexadecyl-sn-glycero-3-phosphocholine	C_24_H_52_NO_6_P	482.3605	21.30	2.37	−0.81	0.0022	0.47
Hydroxyeicosatetraenoic acid	C_20_H_32_O_3_	319.2279	21.53	2.01	−0.60	0.0152	0.66
Monoethylhexyl phthalic acid	C_16_H_22_O_4_	279.1591	22.02	1.74	0.59	0.0152	1.50
LysoPE(18:0/0:0)	C_23_H_48_NO_7_P	480.3096	22.52	1.94	−0.83	0.0022	0.54
MG(18:2/0:0/0:0)	C_21_H_38_O_4_	355.2843	23.04	1.16	−0.58	0.0411	0.13
Hydroxyhexadecanoic acid	C_16_H_32_O_3_	271.2279	23.26	1.35	−0.78	0.0152	0.44
FA 22:6	C_22_H_32_O_2_	327.2330	23.4	2.53	−0.64	0.0087	0.67
FA 16:1	C_16_H_30_O_2_	253.2173	23.58	3.25	−0.71	0.0043	0.62
FA 20:4	C_20_H_32_O_2_	303.2330	23.66	3.28	−0.66	0.0152	0.64
FA 22:5	C_22_H_34_O_2_	329.2486	23.84	1.57	−0.79	0.0022	0.54
FA 18:2	C_18_H_32_O_2_	279.2330	23.91	8.28	−0.81	0.0043	0.51
MG(16:0/0:0/0:0)	C_19_H_38_O_4_	331.2843	24.04	1.15	0.81	0.0022	2.28
FA 20:3	C_20_H_34_O_2_	305.2486	24.32	1.14	−0.76	0.0043	0.57
Hydroxyoctadecanoic acid	C_18_H_36_O_3_	299.2592	24.86	1.03	−0.87	0.0022	0.42
FA 18:1	C_18_H_34_O_2_	281.2486	25.13	9.23	−0.84	0.0022	0.54
FA 20:2	C_20_H_36_O_2_	307.2643	25.41	1.13	−0.79	0.0043	0.54
MG(18:0/0:0/0:0)	C_21_H_42_O_4_	381.2975	25.87	1.98	0.76	0.0260	2.88
FA 20:1	C_20_H_38_O_2_	309.2799	27.27	2.41	−0.86	0.0022	0.49

#### High-dose Cd^2+^ exposure results in 39 differential metabolites in mouse skin tissues

**Figure 3:**
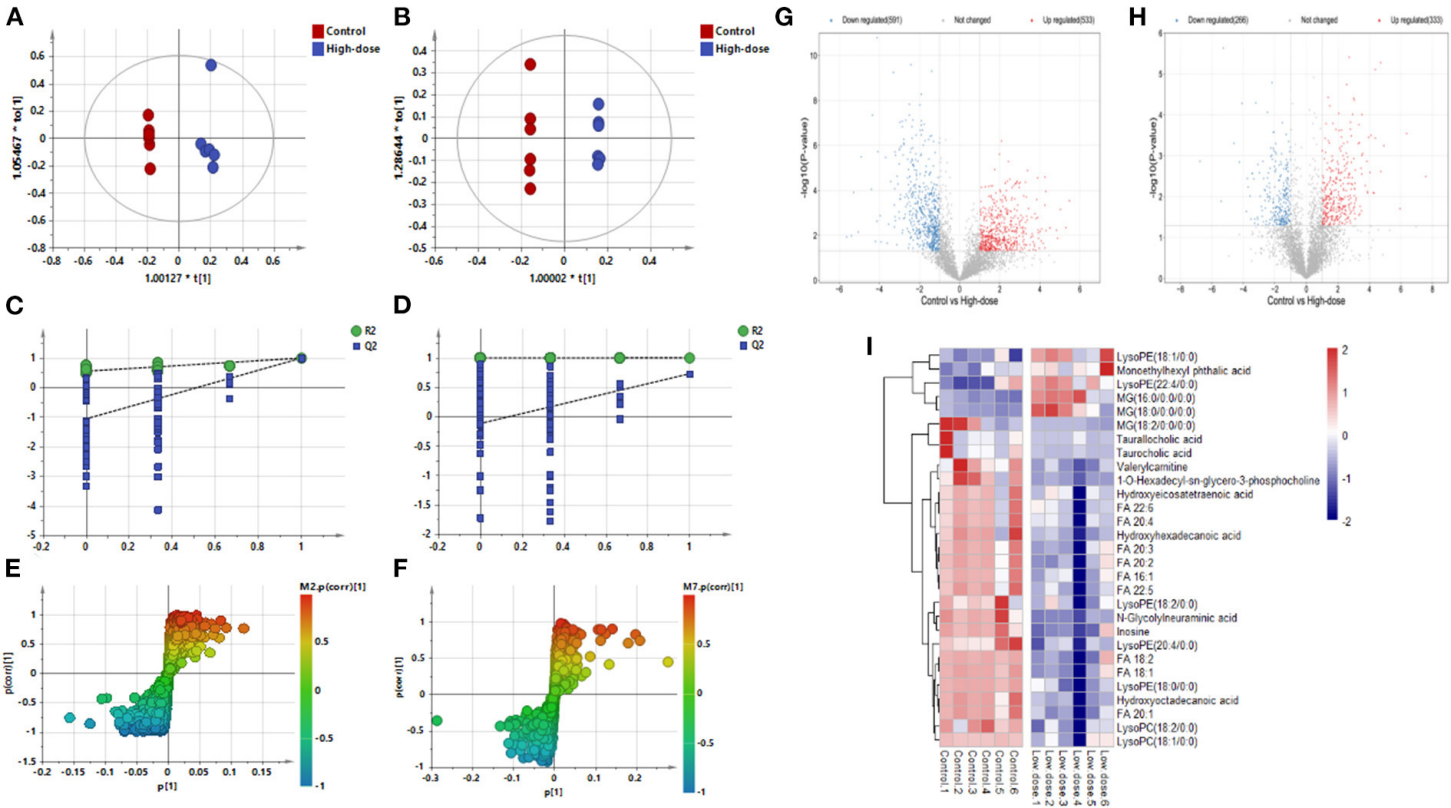
OPLS-DA: Control vs. High-dose. **(A)** Negative ions (R^2^X 0.652, R^2^Y 0.989, Q^2^ 0.898), **(B)** Positive ions (R^2^X 0.787, R^2^Y 1.000, Q^2^ 0.730), **(C)** Negative ion replacement test, **(D)** Positive ion replacement tests, **(E)** Negative ion S-plot, **(F)** Positive ion S-plot. **(G,H)** Volcano diagram. **(G)** Negative ions, **(H)** Positive ions. ① Fold change (M/C) > 2 or <0.5, and ② *P* < 0.05 was the effective variable. Blue means down, red means up. **(I)** Control vs. High-dose differential metabolite heat map. The darker the red, the higher the response, and the darker the blue, the lower the response. It can be seen that the response of most compounds decreased significantly after the administration of low concentrations.

Figures 3A–F shows the OPLS-DA of Control *vs*. High-dose, showing that the two groups are distinguished and the model is valid. Figures 3G,H is a volcano plot of Control vs. High-dose, with red indicating variables up-regulated after CdCl_2_ gavage, blue indicating variables down-regulated after CdCl_2_ gavage, and gray indicating variables that did not differ. A total of 39 different metabolites of Control vs. High-dose were screened and identified. In addition to lysophospholipids, fatty acids, and bile acids, there were also many amino acids, the results are shown in Table 2. The heat map can be more intuitive to observe the difference between the two groups of variable intensity responses, the results are shown in Figure 3I.

**Table 2:** Basic information of differential metabolites in Control vs. High-dose.

**Metabolites**	**Formula**	**m/z**	**Rt min**	**VIP**	***p* (corr)**	***p*-value**	**Fold**
L-Lysine	C_6_H_14_N_2_O_2_	147.1128	1.11	1.27	−0.78	0.0152	0.76
L-Arginine	C_6_H_14_N_4_O_2_	175.1190	1.24	1.76	−0.86	0.0022	0.57
L-Glutamine	C_5_H_10_N_2_O	115.0866	1.25	1.25	−0.84	0.0043	0.60
Anserine	C_10_H_16_N_4_O_3_	241.1295	1.26	1.27	−0.64	0.0260	0.37
Glycerophosphocholine	C_8_H_20_NO_6_P	258.1101	1.27	2.68	−0.70	0.0022	0.33
L-Valine	C_5_H_11_NO_2_	118.0863	1.42	2.15	−0.87	0.0022	0.74
Xanthosine	C_10_H_12_N_4_O_6_	285.0830	4.67	1.61	−0.76	0.0022	0.41
Valerylcarnitine	C_12_H_23_NO_4_	246.1700	9.66	1.30	−0.69	0.0152	0.50
Taurocholic acid	C_26_H_45_NO_7_S	533.3255	15.19	4.81	−0.43	0.0411	0.15
9,10,13-TriHOME	C_18_H_34_O_5_	329.2333	15.96	1.67	0.61	0.0087	2.17
Allocholic acid	C_24_H_40_O_5_	426.3214	17.66	1.81	−0.52	0.0411	0.11
LysoPE(18:2/0:0)	C_23_H_44_NO_7_P	476.2783	19.77	1.87	−0.56	0.0411	0.79
LysoPE(22:6/0:0)	C_27_H_44_NO_7_P	526.2928	19.81	6.19	0.74	0.0043	1.35
LysoPE(20:4/0:0)	C_25_H_44_NO_7_P	502.2928	19.82	5.28	0.71	0.0152	1.24
LysoPE(18:2/0:0)	C_23_H_44_NO_7_P	476.2783	20.03	1.63	−0.72	0.0152	0.71
LysoPC(18:2/0:0)	C_26_H_50_NO_7_P	564.3307	20.13	2.08	−0.70	0.0411	0.80
LysoPE(20:3/0:0)	C_25_H_46_NO_7_P	504.3085	20.43	1.08	0.62	0.0411	1.50
LysoPE(16:0/0:0)	C_21_H_44_NO_7_P	452.2783	20.7	2.21	−0.69	0.0152	0.80
LysoPE(18:1/0:0)	C_23_H_46_NO_7_P	480.3085	20.90	5.72	0.83	0.0022	1.48
LysoPC(18:1/0:0)	C_26_H_52_NO_7_P	544.3374	20.98	1.41	−0.76	0.0152	0.74
LysoPC(16:0/0:0)	C_24_H_50_NO_7_P	496.3398	21.04	3.97	0.91	0.0022	2.00
LysoPE(22:4/0:0)	C_27_H_48_NO_7_P	530.3241	21.13	3.59	0.83	0.0022	2.60
LysoPE(P-16:0/0:0)	C_21_H_44_NO_6_P	438.2979	21.24	3.34	−0.67	0.0087	0.70
1-O-Hexadecyl-sn-glycero-3-phosphocholine	C_24_H_52_NO_6_P	482.3605	21.30	3.06	−0.80	0.0043	0.49
Hydroxyeicosatetraenoic acid	C_20_H_32_O_3_	319.2279	21.53	1.68	−0.59	0.0411	0.79
LysoPE(P-18:1(9Z)/0:0)	C_23_H_46_NO_6_P	462.2990	21.69	1.68	−0.89	0.0043	0.63
LysoPC(19:1/0:0)	C_27_H_54_NO_7_P	536.3711	21.77	3.02	0.90	0.0022	2.22
LysoPC(P-18:0/0:0)	C_26_H_54_NO_6_P	508.3762	21.78	1.76	−0.58	0.0411	0.61
LysoPC(19:1/0:0)	C_27_H_54_NO_7_P	536.3711	22.09	3.22	0.89	0.0022	2.61
LysoPE(18:0/0:0)	C_23_H_48_NO_7_P	482.3241	22.49	2.39	−0.77	0.0022	0.59
Hydroxyhexadecanoic acid	C_16_H_32_O_3_	271.2279	23.26	1.64	−0.81	0.0043	0.45
FA 22:6	C_22_H_32_O_2_	327.2330	23.4	2.70	−0.76	0.0087	0.73
FA 20:4	C_20_H_32_O_2_	303.2330	23.66	3.49	−0.79	0.0087	0.71
FA 22:5	C_22_H_34_O_2_	329.2486	23.84	1.81	−0.89	0.0022	0.61
FA 18:2	C_18_H_32_O_2_	279.2330	23.91	8.29	−0.87	0.0022	0.69
FA 20:3	C_20_H_34_O_2_	305.2486	24.32	1.17	−0.80	0.0152	0.70
Hydroxyoctadecanoic acid	C_18_H_36_O_3_	299.2592	24.86	1.32	−0.94	0.0022	0.40
FA 20:2	C_20_H_36_O_2_	307.2643	25.41	1.12	−0.73	0.0260	0.71
13-Docosenamide	C_22_H_43_NO	338.3417	28.42	2.80	−0.70	0.0087	0.64
L-Lysine	C_6_H_14_N_2_O_2_	147.1128	1.11	1.27	−0.78	0.0152	0.76

#### High- and low-dose Cd exposure triggers differential metabolites in mouse skin tissues

**Figure 4:**
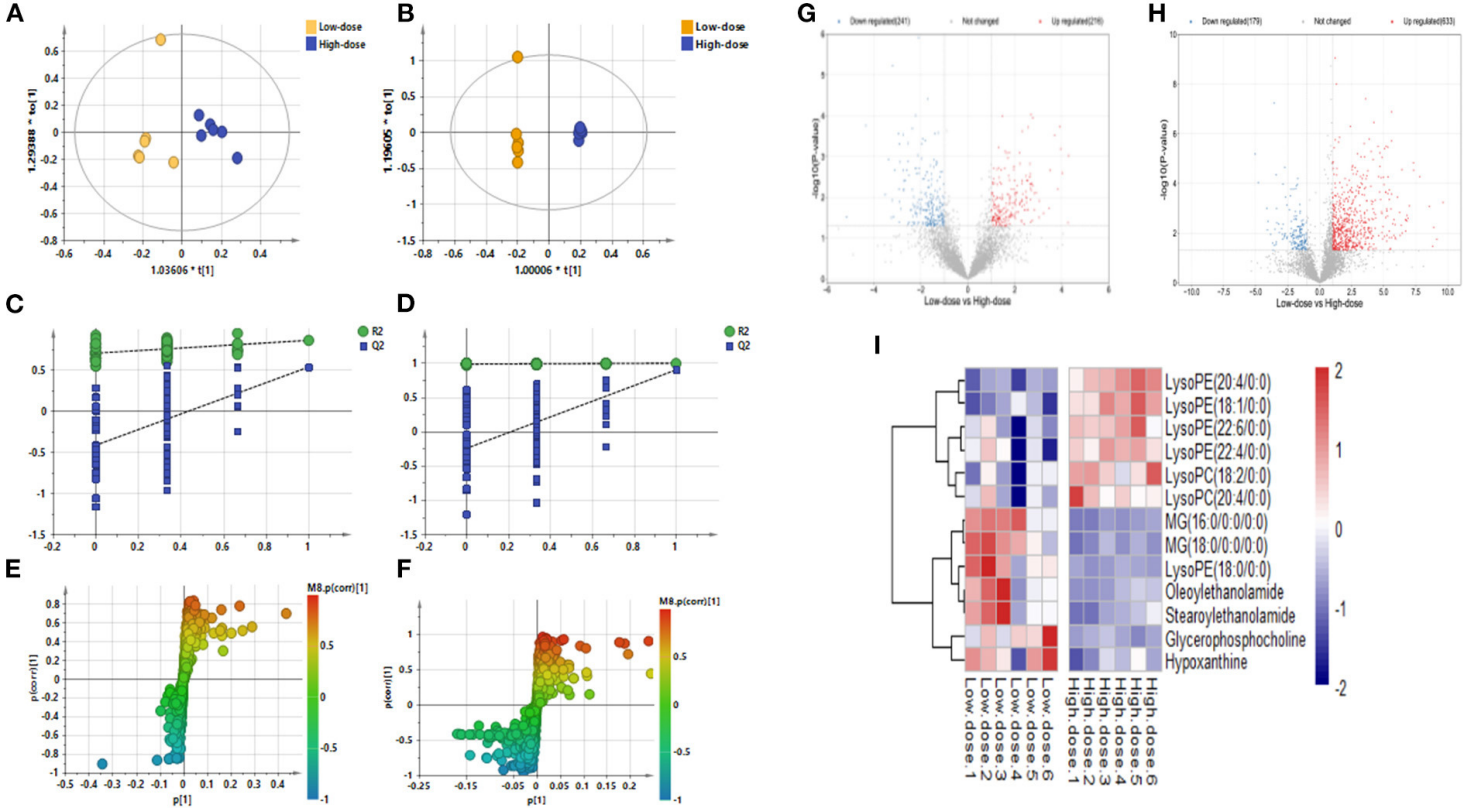
OPLS-DA: Low-dose vs. High-dose. **(A)** Negative ions (R^2^X 0.562, R^2^Y 0.860, Q^2^ 0.536), **(B)** Positive ions (R^2^X 0.874, R^2^Y 0.999, Q^2^ 0.901), **(C)** Negative ion replacement test, **(D)** Positive ion replacement tests, **(E)** Negative ion S-plot, **(F)** Positive ion S-plot. **(G,H)** Volcano diagram. **(G)** Negative ions, **(H)** Positive ions. ① Fold change (M/C) > 2 or <0.5, and ② *P* <0.05 was the effective variable. Blue means down, red means up. **(I)** Low-dose vs. High-dose differential metabolite heat map. The darker the red, the higher the response, and the darker the blue, the lower the response. It can be seen that the response of most compounds decreased significantly after the administration of low concentrations.

Figures 4A–F shows the OPLS-DA plot of Low-dose *vs*. High-dose, with OPLS-DA at negative ions showing relatively small differences between the two groups. Figures 4G,H is a volcano plot of Low-dose vs. High-dose, with red indicating variables up-regulated after CdCl_2_ gavage, blue indicating variables down-regulated after CdCl_2_ gavage, and gray indicating variables that did not differ. Thirteen different metabolites of Low-dose vs. High-dose were screened and identified. Most of them were phospholipids, and the High-dose group had a higher response. The results are shown in Table 3. The heat map can be more intuitive to observe the difference between the two groups of variable intensity responses, the results are shown in Figure 4I.

**Table 3:** Basic information of differential metabolites in Low-dose vs. High-dose.

**Metabolites**	**Formula**	**m/z**	**Rt min**	**VIP**	***p* (corr)**	***p*-value**	**Fold**
Glycerophosphocholine	C_8_H_20_NO_6_P	258.1101	1.27	1.76	−0.68	0.0022	0.42
Hypoxanthine	C_5_H_4_N_4_O	137.0458	4.18	1.18	−0.61	0.0411	0.59
LysoPE(22:6/0:0)	C_27_H_44_NO_7_P	526.2928	19.81	5.65	0.73	0.0043	1.57
LysoPE(20:4/0:0)	C_25_H_44_NO_7_P	502.2928	19.82	6.66	0.91	0.0022	1.69
LysoPC(18:2/0:0)	C_26_H_50_NO_7_P	520.3398	20.14	1.01	0.70	0.0152	1.41
LysoPC(20:4/0:0)	C_28_H_50_NO_7_P	544.3398	20.15	2.16	0.58	0.0411	1.46
LysoPE(18:1/0:0)	C_23_H_46_NO_7_P	480.3085	20.90	5.27	0.88	0.0022	1.75
LysoPE(22:4/0:0)	C_27_H_48_NO_7_P	530.3241	21.13	1.53	0.62	0.0152	1.26
LysoPE(18:0/0:0)	C_23_H_48_NO_7_P	482.3241	22.49	3.11	−0.74	0.0087	0.35
Oleoylethanolamide	C_20_H_39_NO_2_	348.2873	23.40	1.36	−0.63	0.0411	0.36
MG(16:0/0:0/0:0)	C_19_H_38_O_4_	331.2843	24.04	1.33	−0.87	0.0022	0.30
Stearoylethanolamide	C_20_H_41_NO_2_	328.3210	24.76	1.89	−0.67	0.0152	0.43
MG(18:0/0:0/0:0)	C_21_H_42_O_4_	381.2975	25.88	1.08	−0.82	0.0022	0.40

#### Different metabolite profiles in different groups

**Figure 5:**
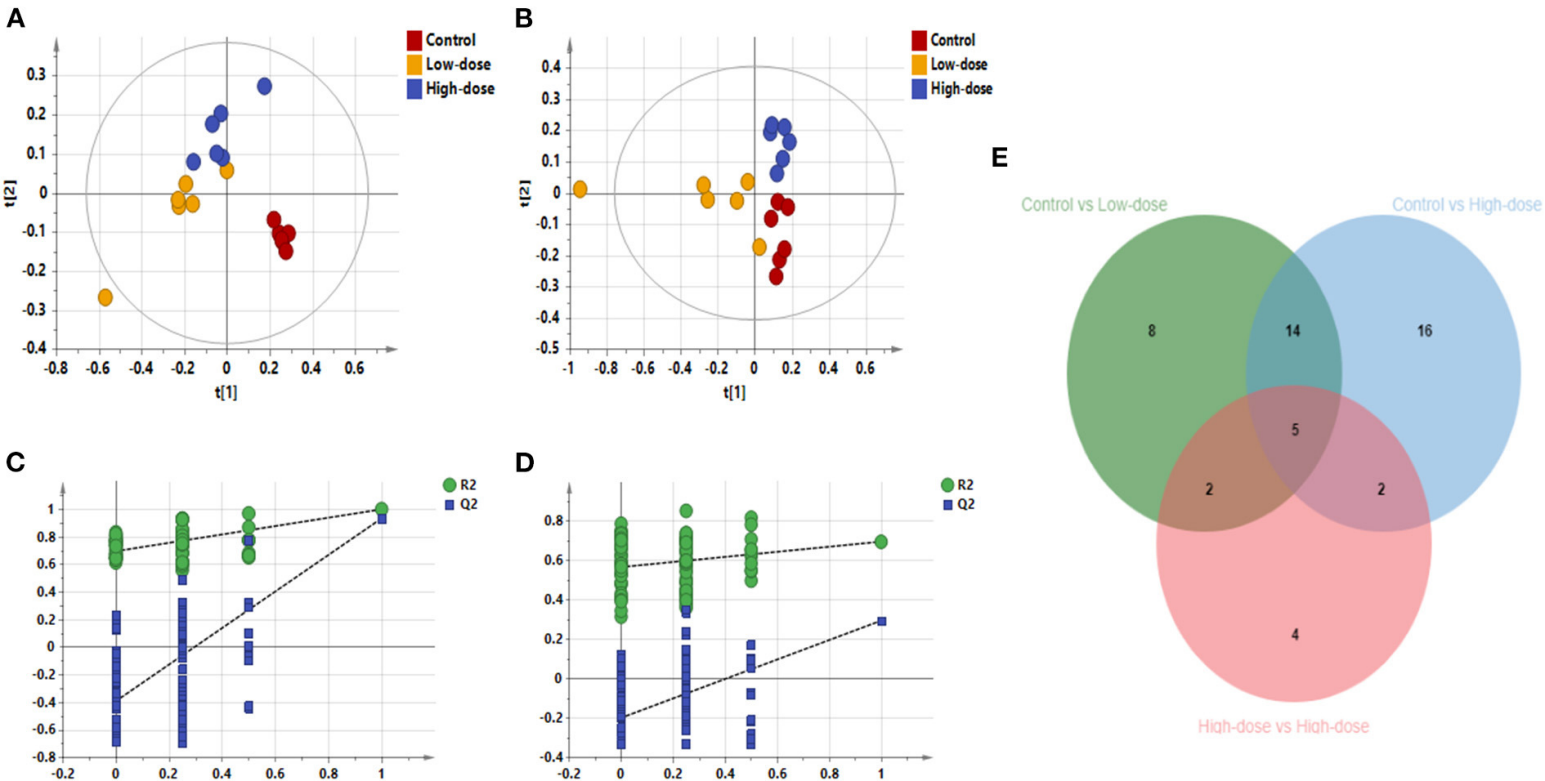
PLS-DA diagram of all samples. **(A)** PLS-DA plot in negative ion mode (R^2^X 0.861, R^2^Y 0.996, *Q*^2^ 0.938), **(B)** PLS-DA plot in positive ion mode (R^2^X 0.636, R^2^Y 0.801, *Q*^2^ 0.559), **(C)** Negative ion replacement test, **(D)** Positive ion replacement test. **(E)** Venn diagram of the number of differential metabolites.

**Figure 6:**
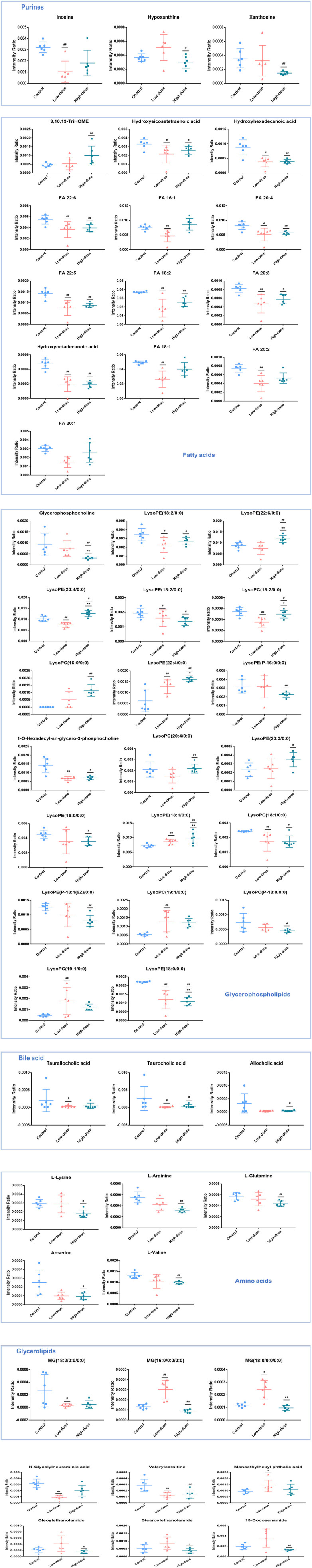
Comparison of three groups of differential metabolites with scatter box plot.

PLS-DA analysis was used to analyze all groups and observe the changing trend of the whole metabolic group of the three groups. Results as shown in Figures 5A–D, the three groups of Control, Low-dose, and High-dose could be distinguished, indicating the migration of the metabolic group after CdCl_2_ administration. In negative ion mode, the distance between the two groups was smaller than that of the control group, suggesting that the effect of CdCl_2_ on the metabolite group may be higher than that of the dose. After pooling all the differential metabolite information involved in the three groups, a total of 53 metabolites were identified, with 5 metabolites being differential metabolites shared by the three models, of which four were lysophosphatidylethanolamine; one was lysophosphatidylcholine, and 49 metabolites showed significant difference after oral administration of CdCl_2_ (Low-dose or High-dose), of which 19 were common metabolites, contains phospholipids, fatty acids, acylcarnitines, bile acids compounds, the results are shown in Table 4. The number of differential metabolites is presented by the Venn diagram, as shown in Figure 5E. The response of each metabolite in three groups was compared by scattering a box plot. The results are shown in Figure 6.

**Table 4:** Classification of differential metabolites.

**No**.	**Name**	**Formula**	**Rt**	**Ion mode**	***cal*m/z**	**m/z**	**ppm**	**MS/MS**	**Class**	**Control vs. Low-dose**	**Control vs. High-dose**	**Low-dose vs. High-dose**
1	L-Lysine	C_6_H_14_N_2_O_2_	1.11	M+H	147.1128	147.1129	0.8	130, 84, 56	Amino acid	-	√	-
2	L-Arginine	C_6_H_14_N_4_O_2_	1.24	M+H	175.1190	175.1191	0.8	175, 130, 116, 70	Amino acid	-	√	-
3	L-Glutamine	C_5_H_10_N_2_O	1.25	M+H	115.0866	115.0872	5.2	130, 84, 56	Amino acid	-	√	-
4	Anserine	C_10_H_16_N_4_O_3_	1.26	M+H	241.1295	241.1298	1.1	241, 170, 153, 126, 109	Amino acid	-	√	-
5	Glycerophosphocholine	C_8_H_20_NO_6_P	1.27	M+H	258.1101	258.1110	3.4	258, 184, 124, 104, 86	Glycerophospholipid	-	√	√
6	N-Glycolylneuraminic acid	C_11_H_19_NO_10_	1.30	M-H	324.0936	324.0949	3.9	324, 236, 186, 116, 87	Sialic acid	√	-	-
7	L-Valine	C_5_H_11_NO_2_	1.42	M+H	118.0863	118.0862	−0.3	72, 55	Amino acid		√	-
8	Inosine	C_10_H_12_N_4_O_5_	3.76	M-H	267.0735	267.0748	5.0	267, 135	Purine	√		-
9	Hypoxanthine	C_5_H_4_N_4_O	4.18	M+H	137.0458	137.0459	0.8	137, 119, 110, 91, 81, 77	Purine	-	-	√
10	Xanthosine	C_10_H_12_N_4_O_6_	4.67	M+H	285.0830	285.0836	2.2	153, 136	Purine		√	-
11	Valerylcarnitine	C_12_H_23_NO_4_	9.66	M+H	246.1700	246.1703	1.4	246, 187, 85	Carnitine	√	√	-
12	Taurallocholic acid	C_26_H_45_NO_7_S	14.01	M+H	516.2990	516.2989	−0.1	533, 516, 498, 480, 462, 337	Bile acid	√	-	-
13	Taurocholic acid	C_26_H_45_NO_7_S	15.19	M+NH_4_	533.3255	533.3260	0.9	533, 516, 498, 480, 462, 337	Bile acid	√	√	-
14	9,10,13-TriHOME	C_18_H_34_O_5_	15.96	M-H	329.2333	329.2345	3.6	329, 229, 211, 171, 139	Fatty acid	-	√	-
15	Allocholic acid	C_24_H_40_O_5_	17.66	M+NH_4_	426.3214	426.3218	1.0	426, 373, 355	Bile acid	-	√	-
16	LysoPE(18:2/0:0)	C_23_H_44_NO_7_P	19.77	M-H	476.2783	476.2785	0.5	476, 279, 214	Glycerophospholipid	√	√	-
17	LysoPE(22:6/0:0)	C_27_H_44_NO_7_P	19.81	M+H	526.2928	526.2937	1.8	526, 385, 354	Glycerophospholipid	-	√	√
18	LysoPE(20:4/0:0)	C_25_H_44_NO_7_P	19.82	M+H	502.2928	502.2936	1.7	502, 361, 330, 287, 269, 203	Glycerophospholipid	√	√	√
19	LysoPE(18:2/0:0)	C_23_H_44_NO_7_P	20.03	M-H	476.2783	476.2790	1.6	476, 279, 196	Glycerophospholipid	-	√	-
20	LysoPC(18:2/0:0)	C_26_H_50_NO_7_P	20.14	M+Na	520.3398	520.3405	1.4	542, 483, 337, 184, 146, 104	Glycerophospholipid	√	√	√
21	LysoPC(20:4/0:0)	C_28_H_50_NO_7_P	20.15	M+H	544.3398	544.3401	0.7	544, 184	Glycerophospholipid	-	-	√
22	LysoPE(20:3/0:0)	C_25_H_46_NO_7_P	20.43	M+H	504.3085	504.3084	−0.1	504, 363	Glycerophospholipid	-	√	-
23	LysoPE(16:0/0:0)	C_21_H_44_NO_7_P	20.70	M-H	452.2783	452.2789	1.4	452, 255, 196	Glycerophospholipid	-	√	-
24	LysoPE(18:1/0:0)	C_23_H_46_NO_7_P	20.90	M+H	480.3085	480.3089	0.9	482, 339, 308	Glycerophospholipid	√	√	√
25	LysoPC(18:1/0:0)	C_26_H_52_NO_7_P	20.98	M+FA-H	566.3463	566.3474	1.9	566, 506, 281	Glycerophospholipid	√	√	-
26	LysoPC(16:0/0:0)	C_24_H_50_NO_7_P	21.04	M+H	496.3398	496.3402	0.9	496, 478, 313, 184, 104	Glycerophospholipid	-	√	-
27	LysoPE(22:4/0:0)	C_27_H_48_NO_7_P	21.13	M+H	530.3241	530.3246	0.9	530, 389	Glycerophospholipid	√	√	√
28	LysoPE(P-16:0/0:0)	C_21_H_44_NO_6_P	21.24	M+H	438.2979	438.2982	0.7	438, 420, 364, 284, 266, 155	Glycerophospholipid	-	√	-
29	1-O-Hexadecyl-sn-glycero-3-phosphocholine	C_24_H_52_NO_6_P	21.30	M+H	482.3605	482.3601	−0.8	482, 184, 104	Glycerophospholipid	√	√	-
30	Hydroxyeicosatetraenoic acid	C_20_H_32_O_3_	21.53	M-H	319.2279	319.2293	4.4	319, 301	Fatty acid	√	√	-
31	LysoPE(P-18:1(9Z)/0:0)	C_23_H_46_NO_6_P	21.69	M-H	462.2990	462.3000	2.2	462, 265, 196, 140, 78	Glycerophospholipid	-	√	-
32	LysoPC(19:1/0:0)	C_27_H_54_NO_7_P	21.77	M+H	536.3711	536.3715	0.8	536, 518, 184	Glycerophospholipid	-	√	-
33	LysoPC(P-18:0/0:0)	C_26_H_54_NO_6_P	21.78	M+H	508.3762	508.3761	−0.1	508, 367, 184, 104	Glycerophospholipid	-	√	-
34	Monoethylhexyl phthalic acid	C_16_H_22_O_4_	22.02	M+H	279.1591	279.1598	2.7	279, 149, 57	Phthalate	√	-	-
35	LysoPC(19:1/0:0)	C_27_H_54_NO_7_P	22.09	M+H	536.3711	536.3715	0.8	536, 518, 184, 104	Glycerophospholipid	-	√	-
36	LysoPE(18:0/0:0)	C_23_H_48_NO_7_P	22.49	M+H	482.3241	482.3246	1.0	482, 464, 421, 341, 310	Glycerophospholipid	√	√	√
37	MG(18:2/0:0/0:0)	C_21_H_38_O_4_	23.04	M+H	355.2843	355.2848	1.6	355, 337, 266, 263, 245	Glycerolipid	√	-	-
38	Hydroxyhexadecanoic acid	C_16_H_32_O_3_	23.26	M-H	271.2279	271.2290	4.3	271, 225	Fatty acid	√	√	-
39	Oleoylethanolamide	C_20_H_39_NO_2_	23.4	M+Na	348.2873	348.2878	1.4	326, 309, 308, 62	Ethanol amine	-	-	√
40	FA 22:6	C_22_H_32_O_2_	23.4	M-H	327.2330	327.2343	4.0	327, 283	Fatty acid, Docosahexaenoic acid	√	√	-
41	FA 16:1	C_16_H_30_O_2_	23.58	M-H	253.2173	253.2187	5.7	253, 126	Fatty acid, Palmitoleic acid	√	-	-
42	FA 20:4	C_20_H_32_O_2_	23.66	M-H	303.2330	303.2347	5.9	303, 259	Fatty acid, Arachidonic acid	√	√	-
43	FA 22:5	C_22_H_34_O_2_	23.84	M-H	329.2486	329.2497	3.3	329, 297, 281, 149	Fatty acid, Docosapentaenoic acid	√	√	-
44	FA 18:2	C_18_H_32_O_2_	23.91	M-H	279.2330	279.2343	4.9	279, 261	Fatty acid, Linoleic acid	√	√	-
45	MG(16:0/0:0/0:0)	C_19_H_38_O_4_	24.04	M+H	331.2843	331.2847	1.3	313, 257, 239, 95, 71, 57	Glycerolipid	√	-	√
46	FA 20:3	C_20_H_34_O_2_	24.32	M-H	305.2486	305.2499	4.4	305	Fatty acid, Eicosatrienoic acid	√	√	-
47	Stearoylethanolamide	C_20_H_41_NO_2_	24.76	M+H	328.3210	328.3219	2.6	328, 62	Ethanol amine	-	-	√
48	Hydroxyoctadecanoic acid	C_18_H_36_O_3_	24.86	M-H	299.2592	299.2597	1.6	299, 253	Fatty acid	√	√	-
49	FA 18:1	C_18_H_34_O_2_	25.13	M-H	281.2486	281.2501	5.3	281, 263	Fatty acid, Oleic acid	√	-	-
50	FA 20:2	C_20_H_36_O_2_	25.41	M-H	307.2643	307.2653	3.6	307	Fatty acid, Eicosadienoic acid	√	√	-
51	MG(18:0/0:0/0:0)	C_21_H_42_O_4_	25.88	M+H	381.2975	381.2981	1.6	341, 281, 267, 109, 95, 57	Glycerolipid	√	-	√
52	FA 20:1	C_20_H_38_O_2_	27.27	M-H	309.2799	309.2811	3.7	309, 291	Fatty acid, Eicosenoic acid	√	-	-
53	13-Docosenamide	C_22_H_43_NO	28.42	M+H	338.3417	338.3423	1.7	338, 321, 303, 177, 149, 135, 83, 69	Fatty amide	-	√	-
**Total**	**29**	**39**	**13**									

### Metabolomics pathway analysis of mouse skin tissues after Cd^2+^ exposure

**Figure 7:**
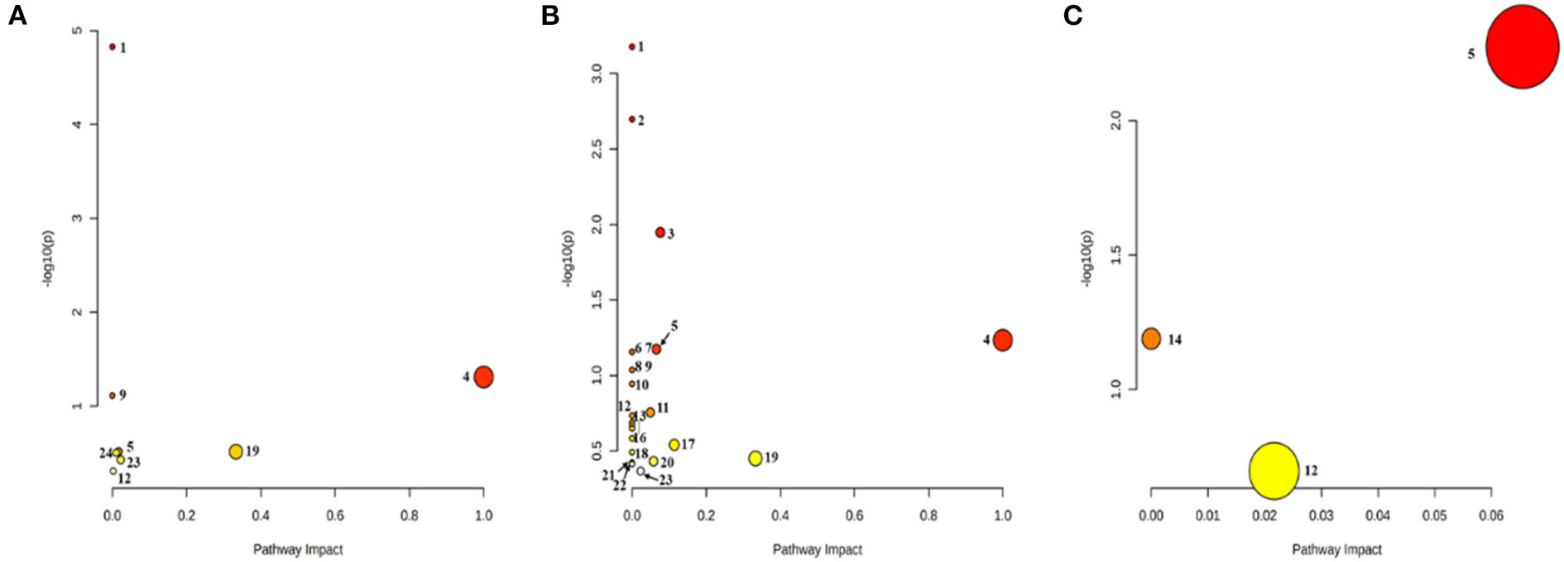
Bubble diagram of the metabolic pathway. **(A)** Control vs. Low-dose; **(B)** Control vs. High-dose; **(C)** Low-dose vs. High-dose. (1) Biosynthesis of unsaturated fatty acids; (2) Aminoacyl-tRNA biosynthesis; (3) Arginine biosynthesis; (4) Linoleic acid metabolism; (5) Glycerophospholipid metabolism; (6) D-Glutamine and D-glutamate metabolism; (7) Nitrogen metabolism; (8) Valine, leucine, and isoleucine biosynthesis; (9) Taurine and hypotaurine metabolism; (10) Biotin metabolism; (11) Histidine metabolism; (12) Purine metabolism; (13) Pantothenate and CoA biosynthesis; (14) Ether lipid metabolism; (15) beta-Alanine metabolisms; (16) Lysine degradation; (17) Alanine, aspartate, and glutamate metabolism; (18) Glyoxylate and dicarboxylate metabolism; (19) Arachidonic acid metabolism; (20) Arginine and proline metabolism; (21) Pyrimidine metabolism; (22) Valine, leucine, and isoleucine degradation; (23) Primary bile acid biosynthesis; (24) Amino sugar and nucleotide sugar metabolism.

The metabolic pathway bubble map shows that the smaller the *p*-value, the larger the bubble, indicating that the metabolic pathway is more significant (the darker the color). According to the types of compounds involved in the metabolic pathways, the main differential compounds in Control vs. Low-dose were Fatty acid metabolism, bile acid metabolism, phospholipid metabolism, taurine metabolism, and purine metabolism. The differential metabolites of Control vs. High-dose involve more amino acid-related metabolism besides the above-mentioned metabolism. Low-dose vs. High-dose involves the metabolism of phospholipids and purines, as shown in Figure 7. The main pathways involved in the different metabolites are summarized in Table 5.

**Table 5:** Metabolic pathway information.

**Pathways**		***p***	**–log(p)**	**Holm *p***	**FDR**	**Impact**	**Details**	**Comments**
**Control vs. Low-dose**								
Fatty acids	Biosynthesis of unsaturated fatty acids	0.0000	4.8286	0.0012	0.0012	0.0000	KEGG	Unsaturated fatty acids
	Linoleic acid metabolism	0.0489	1.3108	1.0000	1.0000	1.0000	KEGG	FA 18:2
	Arachidonic acid metabolism	0.3056	0.5149	1.0000	1.0000	0.3329	KEGG SMP	FA 20:4
Glycerophospholipids	Glycerophospholipid metabolism	0.3056	0.5149	1.0000	1.0000	0.0174	KEGG	LPC; LPE
Bile acids	Taurine and hypotaurine metabolism	0.0771	1.1128	1.0000	1.0000	0.0000	KEGG SMP	Taurocholic acid
	Primary bile acid biosynthesis	0.3735	0.4278	1.0000	1.0000	0.0229	KEGG SMP	Taurocholic acid; Taurallocholic acid
Sialic acid	Amino sugar and nucleotide sugar metabolism	0.3126	0.5050	1.0000	1.0000	0.0106	KEGG SMP SMP	N-Glycolylneuraminic acid
Purines	Purine metabolism	0.4911	0.3089	1.0000	1.0000	0.0025	KEGG SMP	Inosine
**Control vs. High-dose**								
Amino acids	Aminoacyl-tRNA biosynthesis	0.0020	2.6983	0.1663	0.0841	0.0000	KEGG	L-Arginine; L-Glutamine; L-Valine; L-Lysine
	Arginine biosynthesis	0.0113	1.9476	0.9251	0.3159	0.0761	KEGG	L-Arginine; L-Glutamine;
	D-Glutamine and D-glutamate metabolism	0.0697	1.1567	1.0000	0.8366	0.0000	KEGG SMP	L-Glutamine
	Nitrogen metabolism	0.0697	1.1567	1.0000	0.8366	0.0000	KEGG	L-Glutamine
	Valine, leucine and isoleucine biosynthesis	0.0919	1.0366	1.0000	0.8579	0.0000	KEGG	L-Valine
	Biotin metabolism	0.1136	0.9446	1.0000	0.9544	0.0000	KEGG SMP	L-Lysine
	Histidine metabolism	0.1758	0.7550	1.0000	1.0000	0.0492	KEGG SMP	L-Anserine
	Lysine degradation	0.2614	0.5826	1.0000	1.0000	0.0000	KEGG SMP	L-Lysine
	Arginine and proline metabolism	0.3704	0.4313	1.0000	1.0000	0.0579	KEGG SMP	L-Arginine
	Valine, leucine and isoleucine degradation	0.3858	0.4137	1.0000	1.0000	0.0000	KEGG SMP	L-Valine
	Glyoxylate and dicarboxylate metabolism	0.3221	0.4920	1.0000	1.0000	0.0000	KEGG	L-Glutamine
	beta-Alanine metabolism	0.2245	0.6488	1.0000	1.0000	0.0000	KEGG SMP	L-Anserine
	Alanine, aspartate and glutamate metabolism	0.2881	0.5405	1.0000	1.0000	0.1138	KEGG SMP	L-Glutamine
	Pantothenate and CoA biosynthesis	0.2053	0.6875	1.0000	1.0000	0.0000	KEGG SMP	L-Valine
	Pyrimidine metabolism	0.3781	0.4224	1.0000	1.0000	0.0000	KEGG SMP	L-Glutamine
Fatty acids	Biosynthesis of unsaturated fatty acids	0.0007	3.1779	0.0558	0.0558	0.0000	KEGG	Unsaturated fatty acids
	Linoleic acid metabolism	0.0584	1.2334	1.0000	0.8366	1.0000	KEGG	FA 18:2
	Arachidonic acid metabolism	0.3547	0.4502	1.0000	1.0000	0.3329	KEGG SMP	FA 20:4
Glycerophospholipids	Glycerophospholipid metabolism	0.0669	1.1747	1.0000	0.8366	0.0655	KEGG	LPC; LPE
	Ether lipid metabolism	0.2150	0.6676	1.0000	1.0000	0.0000	KEGG	Glycerophosphocholine
Bile acids	Taurine and hypotaurine metabolism	0.0919	1.0366	1.0000	0.8579	0.0000	KEGG SMP	Taurocholic acid
	Primary bile acid biosynthesis	0.4297	0.3668	1.0000	1.0000	0.0229	KEGG SMP	Taurocholic acid; Allocholic acid
Purines	Purine metabolism	0.1849	0.7332	1.0000	1.0000	0.0000	KEGG SMP	Xanthosine; L-Glutamine
**Low-dose vs. High-dose**								
Glycerophospholipids	Glycerophospholipid metabolism	0.0053	2.2747	0.4462	0.4462	0.0655	KEGG	LPC; LPE
	Ether lipid metabolism	0.0647	1.1888	1.0000	1.0000	0.0000	KEGG	Glycerophosphocholine
Purines	Purine metabolism	0.2010	0.6968	1.0000	1.0000	0.0217	KEGG SMP	Hypoxanthine

### Cd^2+^ exposure affects cell proliferation, apoptosis, and hair follicle stem cell content in skin tissue

**Figure 8:**
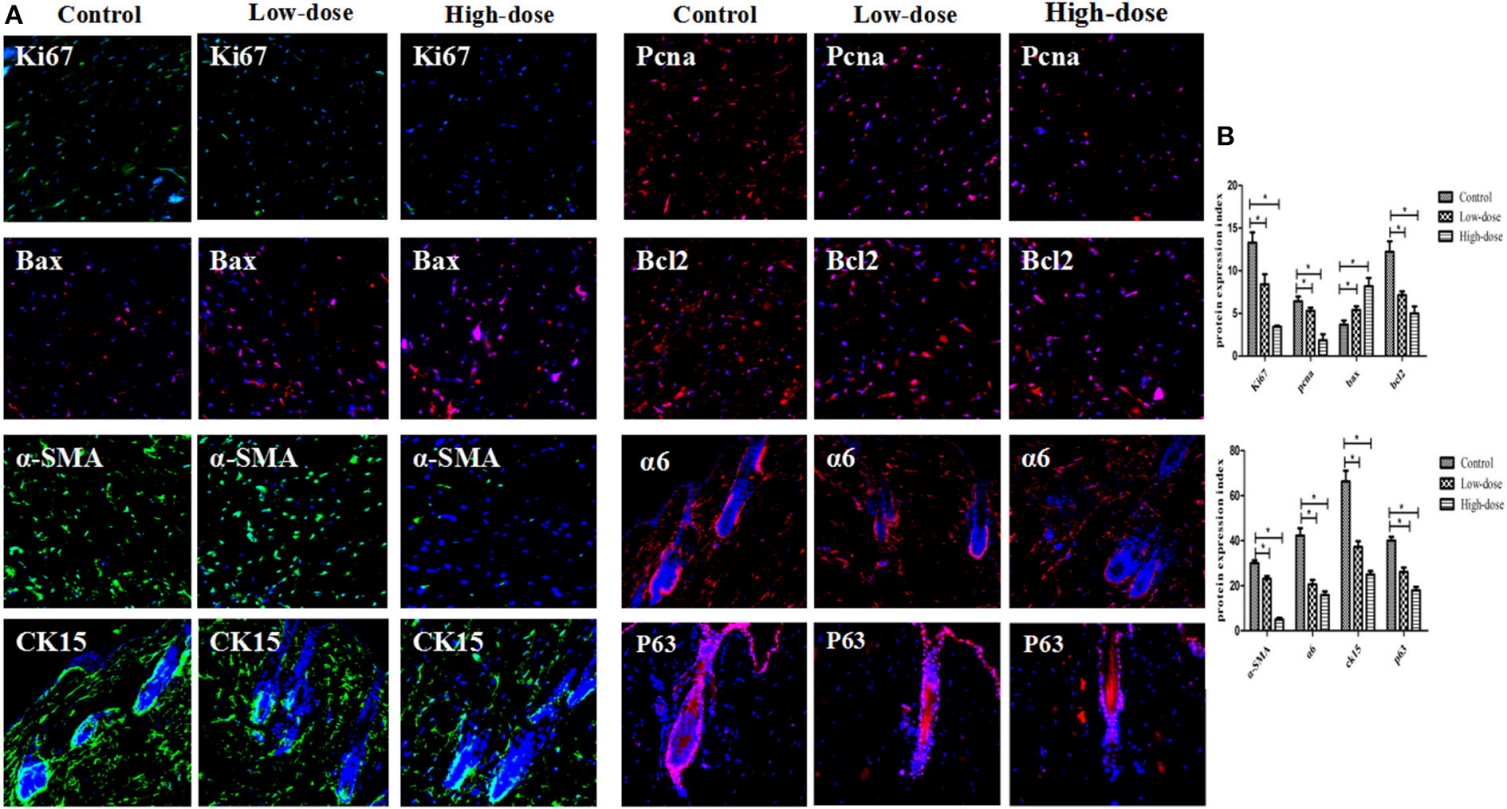
**(A)** Immunofluorescence detection of markers in different groups (X200). **(B)** Comparison of immunofluorescence gray values of markers in different groups. Data are represented as means ± SD (*P < 0.05 vs. control).

**Figure 9:**
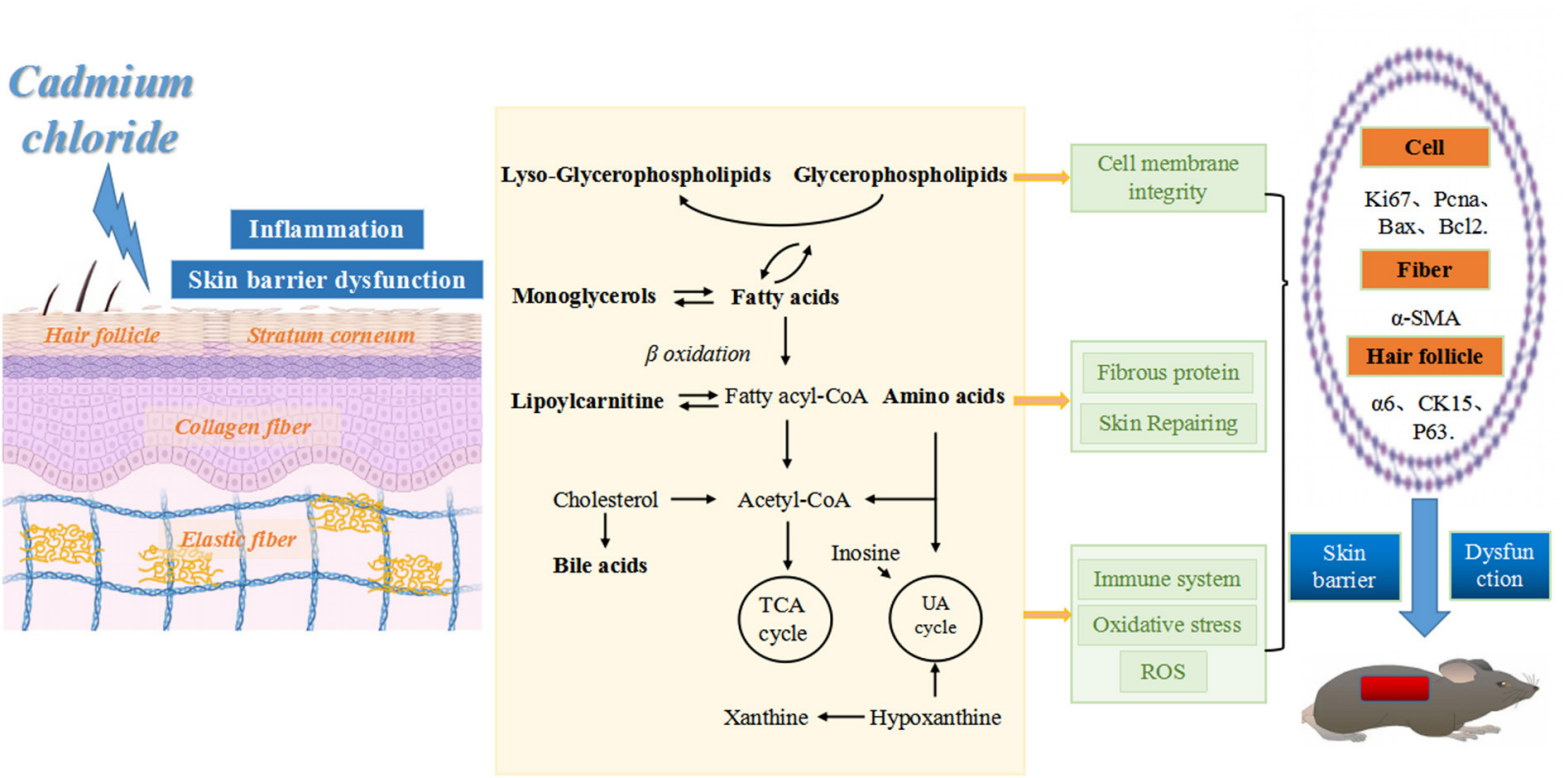
Schematic diagram of skin tissue damage caused by CdCl_2_ gavage. From left to right: CdCl_2_ enters the body and acts on all layers of skin tissue, inducing inflammation and impairment of skin barrier function. This action may occur through the following pathways: (1) affecting the balance of phospholipid metabolism, disrupting the integrity of cell membranes and promoting the inflammatory process, leading to inhibition of cell proliferation (reduced Ki67 and Pcna expression) and accelerated apoptosis (increased Bax expression and reduced Bcl2 expression). (2) Affects amino acid metabolic balance, disrupts collagen fibers (decreased α-SMA expression) and skin repair functions. (3) Affects the metabolic balance of fatty acids, amino acids, bile acids and purines, leading to disruption of energy metabolism, increased oxidative stress, impaired skin immune function and destruction of hair follicle structure (decreased expression of α6, CK15, and P63).

Immunofluorescence staining of skin tissue samples from three groups showed that the expression levels of cell proliferation markers Ki67 (green fluorescence) and Pcna (red fluorescence) decreased with increasing intervention concentrations. The expression of apoptosis marker Bax (red fluorescence) increased with the increase of intervention concentration, and the expression of anti-apoptosis marker Bcl-2 (red fluorescence) decreased with the increase of intervention concentration. The expression of smooth muscle marker α-SMA (green fluorescence) also decreased with the increase of intervention concentration. In the perifollicular region, the Low-dose group showed that the expression positions of the follicular stem cell markers α6 (red fluorescence), Ck15 (green fluorescence), and P63 (red fluorescence) were separated from those of the Control group, and the expression levels were decreased. In the High-dose group, the expression of hair follicle stem cell markers was further disrupted, as shown in Figures 8A,B. Based on metabolic pathway and immunofluorescence analysis, combined with KEGG and literature review results, a schematic diagram was drawn (Figure 9) to investigate the potential mechanism of the effect of CdCl_2_ gavage on skin damage.

## Discussion

Both endogenous and exogenous factors may contribute to skin barrier dysfunction, which may manifest as various symptoms such as skin dryness, ichthyosis, and atopic dermatitis. The lipid sheet is part of the stratum corneum, which is made up of ceramide, cholesterol and fatty acids. The stratum corneum plays a primary protective role in the barrier function, and internal water loss and invasion by microorganisms or exogenous pathogens can be prevented (18, 19). The destruction of the cuticle structure may directly affect the barrier function of the skin. Fatty acids and amino acids are important nutrients in the body, which can metabolize to form acetyl CoA and participate in energy metabolism. The chemical systems of the tricarboxylic acid cycle and respiratory chains are important components of energy metabolism and occur mainly in the mitochondria. Disorders of mitochondrial function and energy metabolism can also lead to oxidative stress and ROS accumulation, which can trigger inflammation (20) and disrupt skin physiological functions. Cd^2+^ is a common pollutant in the environment, more studies are needed to study the comprehensive toxic effects of Cd^2+^. Cd^2+^ has toxic effects on the liver, kidney, brain, skin as well as other organs, and oxidative stress is crucial to its toxic effects (1, 21–23).

In this study, we found that both the Low-dose group (CdCl_2_ 2 mg/kg) and the High-dose group (CdCl_2_ 7 mg/kg) caused metabolic disorders in normal skin tissues of mice. Importantly, low dose Cd^2+^ exposure impaired 29 metabolites in normal skin tissues of mice, while high-dose Cd^2+^ exposure increased another 10 metabolites, suggesting that High-dose Cd^2+^ exposure was more destructive to skin tissue metabolism. These changed metabolites were mainly concentrated in lysophospholipids, fatty acids, bile acids, and more amino acid compounds. And when the three groups of Control, Low-dose, and High-dose were compared simultaneously, five common differential metabolites were found, four of which were lysophosphatidylethanolamine and one was lysophosphatidylcholine, showing the importance of lysophospholipid metabolites in Cd^2+^ exposure damage to skin tissues. Further analysis of metabolic pathways revealed that phospholipid metabolism, fatty acid metabolism, bile acid metabolism and amino acid metabolism were important metabolic pathways in Cd^2+^-exposed damaged skin, which was consistent with the combination of previous differential metabolite analysis. Besides, taurine metabolism and purine metabolism were also involved in this process. In the cell phenotype analysis, with the increase of Cd^2+^ exposure dose, the cell proliferation in the skin tissue was slower, the apoptosis was faster, and the damage to hair follicle stem cells was more significant. This suggests that Cd^2+^ exposure may cause accelerated aging of skin tissues and inhibit hair regeneration. Thus, our study strengthens the understanding of the damage to skin tissues by Cd^2+^ exposure, provides an experimental basis for its study of potential damage mechanisms, and serves as a warning of the hazards of Cd^2+^ exposure in the environment.

The main structural element of a cell membrane is phospholipids. Phospholipids (PLs) represent an important class of biomolecules, of which Glycerophospholipid (GPLs) may play the most important role. Phospholipids are composed of the hydrophobic end of fatty acids and the hydrophilic end of substituted phosphoric acids. They are responsible not only for the organization of cell membranes but also for cell signaling, phospholipids are hydrolyzed by phospholipase into Lyso-PLs and fatty acids (24). Phospholipases are found in neutrophils, which not only secrete phospholipases but also produce ROS, a reaction that may also trigger the production of Lyso-PLs. UV radiation has been found to produce ROS causing oxidative stress, which alters cellular molecules leading to skin barrier dysfunction (25). To ward off harmful oxidation products, skin cells produce detoxifying enzymes and antioxidants. Long-wavelength ultraviolet (UVA-1) oxidation of lipids can activate NRF2-dependent antioxidant pathways (26). Sphingomyelin-containing lecithin can reduce the damage to the skin barrier and relieve skin aging by reducing the ROS level in hairless mice after UV irradiation. Our study found that phospholipids, especially lysophospholipid-like metabolites and metabolic pathways, responded less to the intervention of CdCl_2_, and the differential metabolites and metabolic pathways were more affected in the High-dose group. It is suggested that Cd^2+^ may affect the balance of phospholipid metabolism (phospholipid deposition and phospholipid over-hydrolysis) by inducing oxidative stress, causing dysfunction, disrupting the integrity of skin cell membranes, promoting the inflammatory process in the skin, leading to inhibition of cell proliferation (decreased Ki67 and Pcna expression) and accelerated apoptosis (increased Bax expression and decreased Bcl2 expression), which may be the exposure damages skin tissue by the first metabolic mechanism of action.

Amino acids are the basic building blocks of proteins, including the skin's most abundant fibrin, such as keratin, collagen, and elastin. Skin condition is closely related to collagen and elastic fibers, and self-repair of skin structure is essential to skin health. Amino acids are important nutrients required to promote wound healing and repair damaged skin, and also contribute to maintaining acid-base balance and moisture in cell layers, such as the stratum corneum, etc. (27). Skin is rich in extracellular matrix such as collagen, which is produced mainly by fibroblasts and protects the body from all kinds of external damage. Essential amino acids play an important role in collagen synthesis in the skin (28). Studies have shown that Branched-chain amino acids as protein components (BCAAs) can also play a role in cell signaling (29). The deficiency of leucine and L-Isoleucine can reduce the synthesis of type I and III collagen in the skin by inhibiting the action of mammalian Sirolimus target protein (mTOR) (30). Our research showed that amino acid-like metabolites and metabolic pathways were reduced in response to Cd^2+^ intervention, and the differential metabolites and metabolic pathways were more affected in the High-dose group. And the expression of smooth muscle marker α-SMA also decreased with the increase of Cd^2+^ intervention concentration. It is suggested that Cd^2+^ may accelerate skin aging by disrupting the skin amino acid metabolic balance, disrupting the skin collagen fiber layer (decreased α-SMA expression), and reducing skin elasticity and skin self-healing, which may be the second metabolic mechanism of action of Cd^2+^ exposure damage to skin tissue.

Mitochondria are one of the major producers of cellular ATP, and mitochondrial dysfunction has been associated with a variety of human diseases (31). Many of the mechanisms leading to skin aging are not fully understood, and mitochondrial function is likely to be part of a complex series of processes that lead to the decline and aging of tissue function. A growing body of research suggests that mitochondrial dysfunction and oxidative stress are key features of aging in various tissues, including the skin (32). Loss of the skin barrier during skin aging increases the risk of infection and affects wound healing. The role of mitochondria in protecting the skin barrier from microorganisms has been found to increase rapidly in glycolysis and ATP production in response to *Staphylococcus aureus* infection on the skin, in response to hypoxia-induced metabolic stress (33). Skin problems are directly related to mitochondrial dysfunction and the passage of complex ROS signals. Oxidative damage induced by mitochondrial ROS production is an important molecular basis for various pathophysiological conditions, including aging and cancer (34). It was found that the metabolism of fatty acids and amino acids is an important pathway of the tricarboxylic acid cycle (35). Our results revealed that fatty acid, bile acid, and amino acid metabolites and metabolic pathways were reduced in response to Cd^2+^ intervention, and the differential metabolites and metabolic pathways were more affected in the High-dose group. Since fatty acid, bile acid, and amino acid metabolism are all important pathways of the tricarboxylic acid cycle, it suggests that Cd^2+^ may have broken the tricarboxylic acid cycle, caused mitochondrial dysfunction, caused elevated ROS levels and the onset of oxidative stress, damaged the skin barrier, reduced the body's immune function, and thus accelerated skin aging. This may be another metabolic mechanism of action for Cd^2+^ exposure damage to skin tissue.

The extensive application of Cd^2+^ in industry results in serious environmental pollution, especially in the water environment, which is toxic to aquatic organisms and human beings (13, 36). We call for a reduction in the discharge of heavy metal wastewater from industry and for more centralized treatment to reduce its toxic effects on humans and aquatic life, in particular the protection of endangered species. Hair growth requires ROS signaling from mitochondrial function, and Cd^2+^ disrupts the antioxidant defense system and promotes the production of lipid oxidation products by increasing ROS production and increasing DNA and protein damage (37). Certain concentrations of antioxidants AntiOxBEN2 and Antioxcin4 can induce endogenous ROS protective pathways in primary human skin fibroblasts (PHSF) (38). There are many kinds of skin cells, hair follicle stem cells are the key factor of hair regeneration, and are the foundation of the formation of functional skin. Our results showed that cell inhibition and apoptosis were most pronounced in the High-dose group, and hair follicle stem cells suffered the most severe damage (expression of α6, CK15, and P63 were all significantly decreased), suggesting that Cd^2+^ may activate ROS signaling, disrupt mitochondrial function, lead to increased oxidative stress, accelerate skin aging and inhibit hair regeneration, and thus exacerbate skin function decreases. This may be one of the molecular mechanisms by which Cd^2+^ exposure damages skin tissue.

## Conclusion

In conclusion, our results show that lysophospholipids, fatty acids, bile acid metabolites, amino acid metabolites, and metabolic pathways changed significantly after CdCl_2_ treatment, it may play a key role in the mechanism of Cd^2+^ breaking the skin barrier. The disruption of metabolic homeostasis such as bile acids and purines may also affect the immune system and the body's thermogenesis. CdCl_2_ inhibited the proliferation of mouse skin cells, promoted cell apoptosis, and accelerated the destruction of hair follicle stem cells. This study provides an experimental basis for predicting the risk of skin harm by Cd^2+^ and exploring the potential mechanism of action and provides strong evidence for the prevention of skin harm by environmental safety. Further studies are needed to elucidate whether changes in the levels of lysophospholipids, fatty acids, bile acid metabolites, and amino acid metabolites following CdCl_2_ intervention can predict skin aging risk and explore potential mechanisms.

## Data availability statement

The original contributions presented in the study are included in the article/supplementary material, further inquiries can be directed to the corresponding authors.

## Ethics statement

The animal study was reviewed and approved by the Zhejiang Chinese Medical University Animal Ethics Committee (No. IACUC-20211227-03).

## Author contributions

WD, ZW, MW, JJ, and SY did the data collection and writing. WD, YD, and ZW were major contributors to writing the manuscript. HR and RQ contributed to the conception and design of the study. All authors read and approved the final manuscript.

## Funding

This work was supported by National Natural Science Foundation of China (No. 81904053), Special Research Project of the Affiliated Hospital of Zhejiang Chinese Medical University (No. 2021FSYYZY43), Hangzhou Medical and Health Technology Planning Project (No. B20220021), Hangzhou Science and Technology Planning Project (No. 2020ZDSJ0042), and Hangzhou Xiaoshan District Science and Technology Planning Project (No. 2019216).

## Conflict of interest

The authors declare that the research was conducted in the absence of any commercial or financial relationships that could be construed as a potential conflict of interest.

## Publisher's note

All claims expressed in this article are solely those of the authors and do not necessarily represent those of their affiliated organizations, or those of the publisher, the editors and the reviewers. Any product that may be evaluated in this article, or claim that may be made by its manufacturer, is not guaranteed or endorsed by the publisher.
